# Expression and Functional Analysis of Hepcidin from Mandarin Fish (*Siniperca chuatsi*)

**DOI:** 10.3390/ijms20225602

**Published:** 2019-11-09

**Authors:** Yawei Shen, Ziwei Zhao, Jinliang Zhao, Xiaowu Chen, Ming Cao, Minglin Wu

**Affiliations:** 1 National Demonstration Center for Experimental Fisheries Science Education, Shanghai Ocean University, Shanghai 201306, China; m1601101058@st.shou.edu.cn (Y.S.); m180100151@st.shou.edu.cn (Z.Z.); 2 Shanghai Engineering Research Center of Aquaculture, Shanghai 201306, China; 3 Shanghai Collaborative Innovation for Aquatic Animal Genetics and Breeding, Shanghai 201306, China; 4 Guangdong Provincial Fishery Germplasm Conservation Center, Guangzhou 511400, China; fishery2009@126.com; 5 Fisheries Research Institute, Anhui Academy of Agricultural Sciences, Hefei 230000, China; mlw2018@163.com

**Keywords:** mandarin fish, liver, hepcidin, iron balance, immune response, miR-19c-5p

## Abstract

Hepcidin is a liver-derived peptide hormone that is related to iron balance and immunity in humans. However, its function in *Siniperca chuatsi* has not been well elucidated. In this study, we analyzed the expression and function of the *S. chuatsi* hepcidin (*Sc-hep*) gene. *Sc-hep* was specifically expressed in the liver and appeared to be one of the most highly expressed genes in the liver. After spleen and kidney necrosis virus (ISKNV) infection and lipopolysaccharide (LPS) and polyinosinic—polycytidylic acid (Poly I:C) stimulation, the expression of *Sc-hep* in the liver increased by approximately 110, 6500, and 225 times, respectively. After ferrous sulfate (FS) injection, the expression of *Sc-hep* in the liver increased approximately 520-fold. We found that miR-19c-5p could inhibit *Sc-hep* expression. Five CpG dinucleotides distributed in the promoter region showed no differential methylation between the liver and the stomach, both presenting high methylation rates. After FS or LPS injection, the expression of three iron balance-related genes (*FPN1*, *TFR1*, and *FTN*) and five immune-related cytokine genes (*IL-1*β, *IL8*, *TNF-*α, *TLR22*, and *SOCS3*) significantly changed. These results indicate that *Sc-hep* participates in the regulation of iron balance and plays an important role in the immune system. *Sc-hep* increased approximately 52-fold when mandarin fish were domesticated with artificial diets. *Sc-hep* might be used as a real-time biomarker of mandarin fish liver because its expression markedly varies under different physiological conditions.

## 1. Introduction

Mandarin fish (*Siniperca chuatsi*) has economic importance in China; it is a carnivorous freshwater fish that is fed exclusively live fish in aquaculture [1]. Artificial feed domestication of mandarin fish in aquaculture is interesting and economically valuable. However, high-density farming causes various diseases, such as infectious spleen and kidney necrosis virus (ISKNV) infection, which causes sudden death of mandarin fish. It is one of the most serious pathogenic factors affecting mandarin fish farming [2].

The liver is mainly involved in nutrition, metabolism, detoxification, and bile secretion for digestion [3,4]. It is also responsible for immunological reactions; specifically, the mononuclear phagocyte system of the liver contains many immunologically active cells, acting as a barrier for antigens carried to the liver via the portal system [5,6]. Therefore, the liver is an organ with multiple functions. Hepcidin is mainly produced by the hepatocytes, is secreted into the serum, and is involved in iron balance and immunity in humans [7]. This peptide was first discovered in 2000 as an antimicrobial protein expressed by the human liver and was named LEAP-1 [8]. This cysteine-rich peptide is also found in human urine and is derived from the liver; since it has antibacterial properties, it was also named hepcidin [9]. Human hepcidin comprises a signal peptide (24 amino acids [aa]), a prodomain (35 aa), and a mature peptide (25 aa). The mature hepcidin is a tightly folded polypeptide with 32% β-sheet structure and four disulfide bonds composed of eight cysteine residues that stabilize the hairpin structure [10]. In fish, hepcidin was first isolated and purified from hybrid striped bass in 2002 [11].

Iron loading can induce hepcidin expression in vivo in mice and humans [12]. Ferroportin 1 (FPN1) [13], transferrin receptor 1 (TFR1) [14], and ferritin (FTN) [15] are three important genes regulating iron balance; hepcidin regulates iron balance by internalizing and degrading FPN1 on the basolateral membrane (BLM) of cells to inhibit iron output, thereby controlling extracellular iron concentration and systemic iron balance [16,17]. Hepcidin expression is also strongly induced during infection and inflammation, and the regulatory role of hepcidin in the inflammatory response is supported by many studies on the pathogenesis of infection [18,19,20]. In mammals, for example, hepcidin contributes to the anti-inflammatory and pro-inflammatory effects of inflammatory cytokines, such as suppressor of cytokine signaling 3 (SOCS3) and tumor necrosis factor alpha (TNF-α) [7]. In fish, such as *Danio rerio* [21], *Ctenopharyngodon idellus* [22], *Sparus aurata* [23], *Pseudosciaena crocea* [24], *Dicentrarchus labrax* [25], *Trachidermus fasciatus* [26], *Brachymystax lenok* [27], and *Salmo trutta* [28], the hepcidin gene has been cloned and proven to participate in the immune response. However, the mechanism by which hepcidin influences the immune function and iron balance in mandarin fish remains unclear.

In this study, we analyzed the molecular characteristics and expression pattern of *S. chuatsi* hepcidin (Sc-hep) and explored its transcription regulation mechanism. Our results provide valuable information about the function of hepcidin in fish.

## 2. Results

### 2.1. Sequence Analysis of Sc-Hep

**Figure 1:**
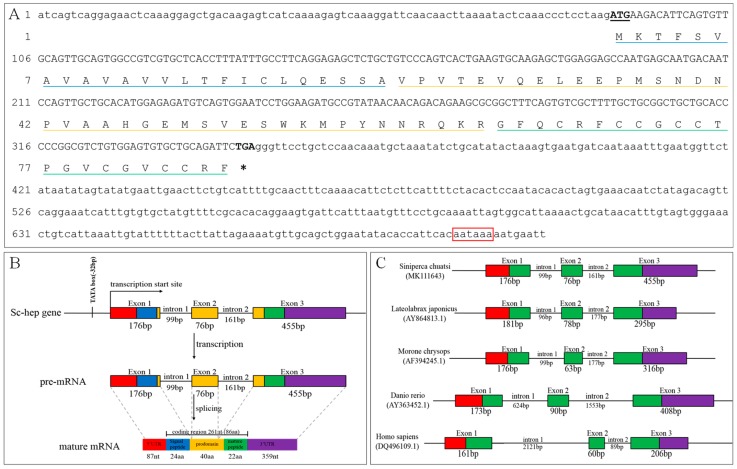
Sequence analysis of Sc-hep. (**A**): cDNA and deduced amino acid sequences of the *Sc-hep* gene. The black underline and the asterisks represent the start and stop codons, respectively. The blue, yellow, and green underlines indicate signal peptide, propeptide, and mature peptide, respectively. The red squares denote the PolyA signal. (**B**): Analysis of *Sc-hep* gene structure. (**C**): Comparison of vertebrate hepcidin gene structure. The boxes, lines, red regions, and purple regions represent exons, introns, 5’-UTR, and 3’-UTR, respectively. The blue, yellow, and green regions represent signal peptides, propeptides, and mature peptides, respectively.

*Sc-hep* mRNA was found to be 707 bp in length and to encode a peptide of 86 aa residues. The 5′- and 3’-UTR sequences aooeared to be 87 and 359 bp in length, respectively. The poly A signal AATAAA was found in the 3’-UTR. Sc-*hep* sequence was submitted to the GenBank database (accession number: MK111643, Figure 1A).

The *Sc-hep* gene appeared to be composed of three exons and two introns, dimilar to those of *Lateolabrax japonicus*, *Morone chrysops*, *D. rerio,* and *Homo sapiens*. However, the introns of *H. sapiens* and *D. rerio* genes are longer than those of *S. chuatsi*, *L. japonicus,* and *M. chrysops* genes (Figure 1B,C).

**Figure 2:**
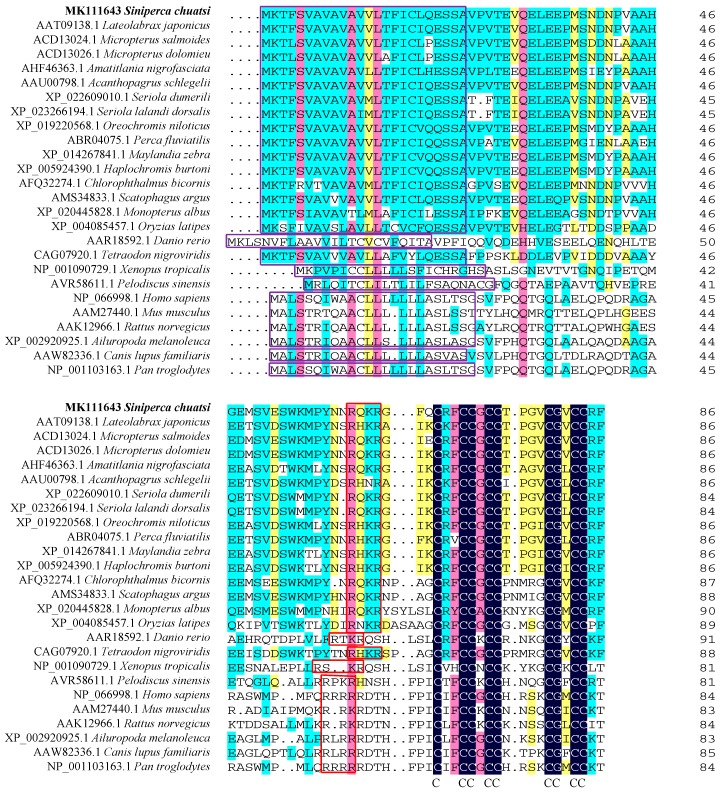
Multiple-sequence alignment of the hepcidin protein. The dark blue regions indicate completely identical amino acid sequences. The pink, cyan, and yellow regions indicate amino acid similarities greater than 75%, 50%, and 33%, respectively. The purple and red squares indicate signal peptide and RX(K/R)R motif, respectively. Mazarine represents the conserved eight cysteine residues.

The first 24 aa form the signal peptide. Comparison with other vertebrate hepcidin aa sequences showed a typical RX(K/R)R motif (R^61^Q^62^K^63^R^64^) in the propeptide that can be recognized by propeptide convertase. The mature peptide comprises amino acids 65 to 86, whose theoretical *pI* and molecular weight are 8.23 and 2.35 kDa, respectively. Eight conserved cysteine residues were found in the mature peptide, consistent with the reslts in other vertebrates. The aa sequence of Sc-hep appeared to be highly identical with that of *Micropterus salmoides* (90%), *Micropterus dolomieu* (90%), *L. japonicus* (88%), and *Acanthopagrus schlegelii* (84%) (Figure 2).

**Figure 3:**
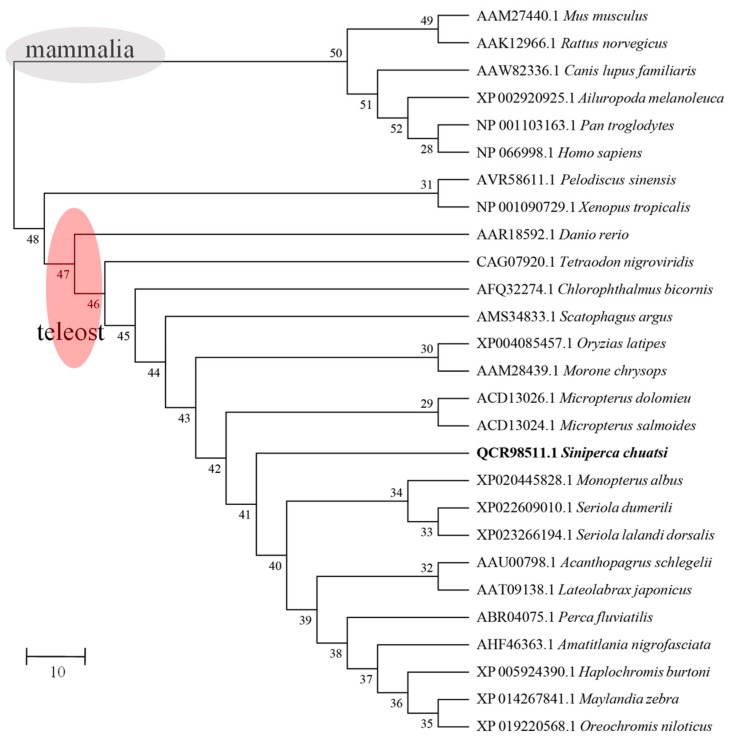
Phylogenetic MP tree based on hepcidin amino acid sequences. A phylogenetic tree based on the hepcidin amino acid sequence of multiple vertebrates was constructed using the Clustal W program and the neighbor-joining method of MEGA 7.0. The node value is a percentage of 1000 replicates.

Phylogenetic analysis showed that *Sc-hep* exhibited high similarity to its homologs from *M. salmoides*, *M. dolomieu*, *L. japonicus*, and *A. schlegelii* (Figure 3). The genetic relationship between species is consistent with their traditional taxonomic status.

**Figure A1:**
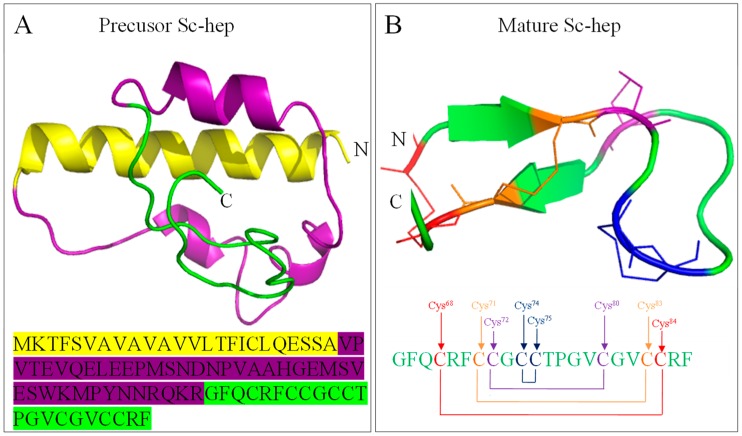
Structure prediction of the Sc-hep protein. (**A**): Protein structure of the precursor Sc-hep. The yellow region is the signal peptide with an α-helix structure. The purple region is the propeptide with a random coil structure. The green region is the mature peptide with a β-sheet structure. (**B**): Structure of the mature Sc-hep protein. Four disulfide bonds are formed by eight cysteine residues to stabilize the whole fold of mature Sc-hep. The connection mode is distinguished by different colors. The red, orange, purple, and blue regions represent Cys68–Cys84, Cys71–Cys82, Cys72–Cys80, and Cys74–Cys75, respectively.

The predicted tertiary protein structure of Sc-hep showed that the signal peptide has an α-helix structure, the propeptide has a random coil structure, and the mature peptide has a β-sheet structure (Figure A1A). The whole folding of mature Sc-hep is stabilized by four disulfide bonds (Figure A1B).

### 2.2. Sc-Hep Expression

**Figure 4:**
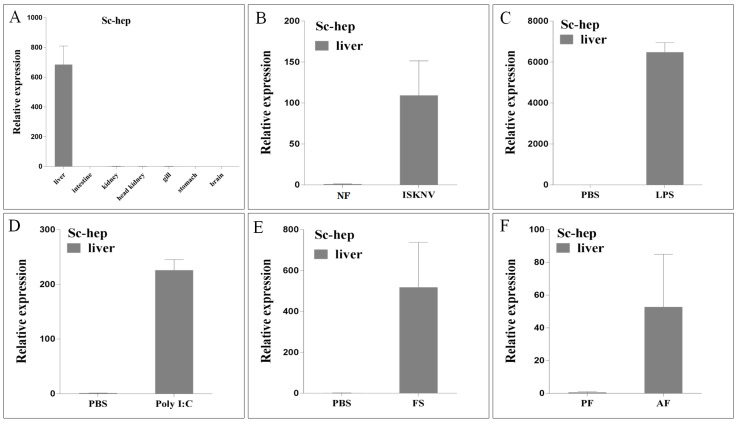
Expression of *Sc-hep*. (**A**): Expression of *Sc-hep* mRNA in seven tissues. (**B**–**E**) and (**F**) show the differential expression of the *Sc-hep* gene in the liver caused by spleen and kidney necrosis virus (ISKNV) infection, lipopolysaccharide (LPS) injection, polyinosinic–polycytidylic acid (Poly I:C) injection, ferrous sulfate (FS) overload, and artificial feed domestication, respectively. AF: fish fed with artificially diet; PBS: phosphate-buffered saline; PF: fish cultured with live prey; NF: normal fish.

*Sc-hep* was found to be expressed in the liver (Figure 4A). In fish naturally infected with ISKNV, stimulated with lipopolysaccharide (LPS) and polyinosinic-polycytidylic acid (Poly I:C), *Sc-hep* expression increased approximately 110-, 6500-, and 225-fold in the liver, respectively (Figure 4B–D). After ferrous sulfate (FS) injection, *Sc-hep* expression increased approximately 520-fold in the liver (Figure 4E). *Sc-hep* expression increased approximately 52-fold when the fish were domesticated with artificial diets (Figure 4F).

### 2.3. Relationship Between Sc-Hep and miR-19c-5p

**Figure 5:**
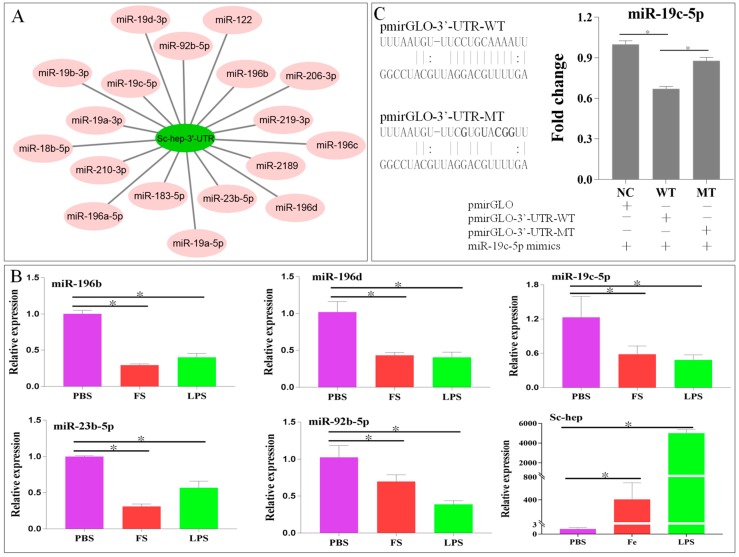
Identification of the targeting relationship between *Sc-hep* and miRNA. (**A**): Prediction of *Sc-hep* targeting by miRNA. (**B**): Expression of five miRNAs negatively correlated with *Sc-hep* expression. (**C**): A dual-luciferase reporter assay was performed to identify the targeting relationship between miR-19c-5p and *Sc-hep*. Statistical analysis results are expressed as means ± SEM; * represents *p* < 0.05, indicating significant differences between the groups.

A total of 18 miRNAs have targeted binding sites in the 3’-UTR *of Sc-hep* mRNA (Figure 5A). The qPCR results showed that expression of miR-196b, miR-196d, miR-23b-5p, miR-92b-5p, and miR-19c-5p were negatively correlated with the expression of *Sc-hep* gene in the liver 24 h after FS or LPS injection (Figure 5B). The relationship between these five miRNAs and *Sc-hep* was verified by a dual-luciferase reporter assay. Only the luciferase activity of wild-type *Sc-hep*-3’-UTR reporter gene co-transfected with miR-19c-5p mimics was significantly reduced compared with that of the negative control group (NC) (*p* < 0.05). The inhibitory activity was abolished after the putative binding sites of miR-19c-5p and *Sc-hep*-3’-UTR were mutated (Figure 5C). These results indicated that miR-19c-5p could target *Sc-hep* 3’-UTR and inhibit its expression.

### 2.4. Sc-Hep DNA Methylation Analysis

**Figure 6:**
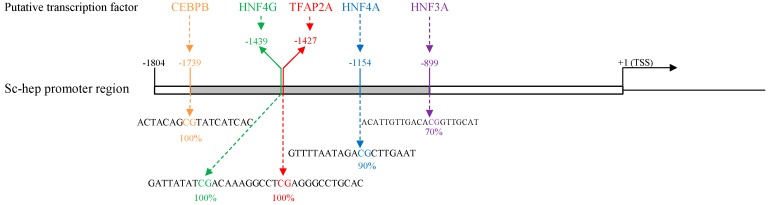
DNA methylation analysis of *Sc-hep*. TSS: transcription start site. The black box indicates *Sc-hep* promoter region. The percentages indicate the methylation level of five CpG sites. The dotted lines denote the CpG sites within the consensus sequence of putative transcription factors.

The 1804 bp promoter region of the *Sc-hep* gene contains several regulatory elements and binding motifs for transcription factors. The TATA box is located at −32 upstream of the transcription start site (TSS), and 17 putative transcription factor bindings sites are distributed in the promoter region (Table A1). Five CpG dinucleotides exist in the promoter at positions −899, −1154, −1427, −1439, and −1739, whose methylation levels were found to be 100%, 100%, 100%, 90%, and 70%, respectively. Liver and stomach tissues did not differ. Transcription factors predicted to bind to *Sc-hep* were identified as CCAAT-enhancer-binding protein-beta (CEBPB), hepatocyte nuclear factor 4 gamma (HNF4G), transcription factor AP-2 alpha (TFAP2A), HNF4A, and HNF3A (Figure 6).

### 2.5. Expression of Iron Balance-Related Genes

Eight genes, namely, ferroportin 1(*FPN1*), transferrin receptor 1 (*TFR1*), ferritin (*FTN1*), interleukin-1 beta (*IL-1β*), interleukin-8 (*IL8*), toll-like receptor 22 (*TLR22*), tumor necrosis factor-alpha (*TNF-α*), and suppressor of cytokine signaling 3 (*SOCS3*), of mandarin fish were also cloned, and their sequences were submitted to the GenBank database (accession numbers: MK605396, MK605398, MK605397, AY647430.1, JN180845.1, JN969981.1, MK605399, and KT895250.1, respectively).

**Figure 7:**
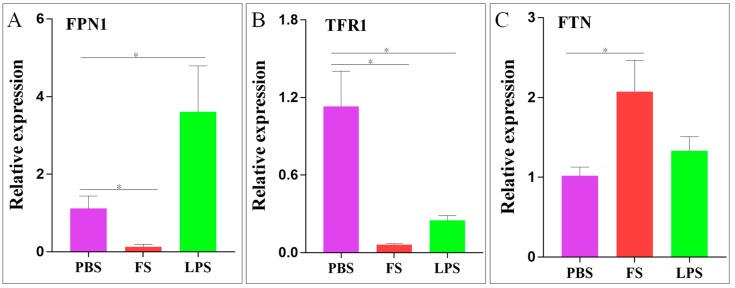
Differential expression of iron balance-related genes in the liver after FS or LPS injection. (**A**): ferroportin 1 (*FPN1*); (**B**): transferrin receptor 1 (*TFR1*); (**C**): ferritin (*FTN*). Statistical analysis results are expressed as means ± SEM; * represents *p* < 0.05, indicating significant differences between the groups.

The mRNA levels of *Sc-hep* and iron balance-related genes (*FPN1*, *TFR1*, and *FTN*) were measured in the liver 24 h post-injection with PBS, FS, or LPS. In the FS injection group, the relative expression levels of *FPN1* and *TFR1* significantly decreased 8.7- and 4.5-fold, respectively. Conversely, the relative expression level of *FTN* was upregulated 2-fold. In the LPS injection group, the relative expression level of *FPN1* significantly increased 3.2-fold, whereas the relative expression level of *TFR1* significantly decreased 18.2-fold. The relative expression of *FTN* was not significantly different (Figure 7).

### 2.6. Expression of Immune-Related Genes

**Figure 8:**
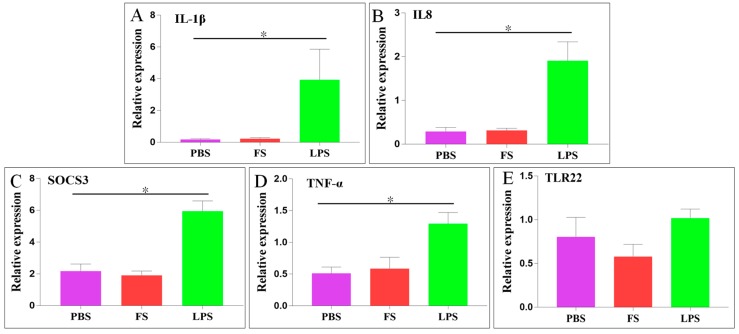
Differential expression of immune-related genes in the liver after FS or LPS injection. (**A**): interleukin-1 beta (*IL-1β*); (**B**): interleukin-8 (*IL8*); (**C**): suppressor of cytokine signaling 3 (*SOCS3*); (**D**): tumor necrosis factor-alpha (*TNF-α*); (**E**): toll-like receptor 22 (*TLR22*). Statistical analysis results are expressed as means ± SEM; * represents *p* < 0.05, indicating significant differences between the groups.

The mRNA levels of immune-related genes (*IL-1β*, *IL8*, *TNF-α*, *TLR22*, and *SOCS3*) in the liver were determined 24 h post-injection with PBS, FS, or LPS. In the LPS group, the relative expression levels of *IL-1β*, *IL8*, *SOCS3*, and *TNF-α* were significantly upregulated 22.3-, 6.6-, 2.7-, and 2.5-fold, respectively, whereas the relative expression of *TLR22* was not significantly different. In the FS group, the relative expression levels of these five genes were not significantly different (Figure 8).

### 2.7. Analysis of Protein–Protein Interactions and Iron Balance Mechanism

**Figure A2:**
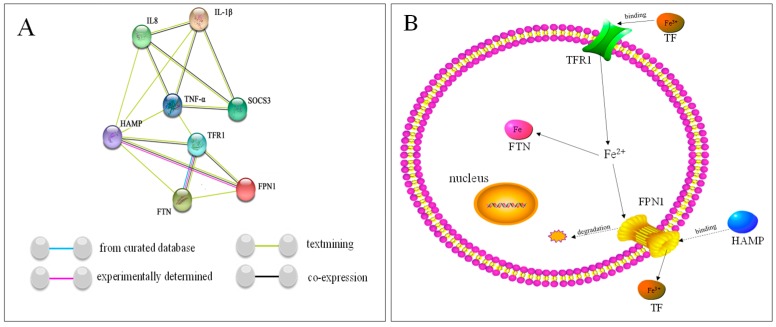
Analysis of iron balance and immune-related genes. (**A**): Protein–protein interaction analysis. The interactions among hepcidin (HAMP), FPN1, TFR1, FTN, IL-1β, IL8, TNF-α, and SOCS3 proteins were analyzed using a human model. The spheres and lines represent proteins and interactions. The light blue, pink, olive, and black lines represent the analysis from the database, experimental data, text mining data, and co-expression data, respectively. (**B**): Model diagram of iron balance mechanism. Transferrin (TF) carries Fe^3+^ and binds to TFR1. In this process, Fe^3+^ is changed to Fe^2+^ by other accessory proteins, and Fe^2+^ is transported into cells by TFR1. FTN can store excess Fe^2+^ in the cells. Fe^2+^ is exported outside of the cells via FPN1. In this process, Fe^2+^ is changed to Fe^3+^ by other accessory proteins. Fe^3+^ is then carried by TF and transported by blood to the site where it is stored or needed. HAMP binds to FPN1, internalizes, and degrades FPN1 to block iron output.

Protein–protein interactions (PPIs) among the eight proteins, namely, hepcidin, FPN1, TFR1, FTN, IL-1β, IL8, TNF-α, and SOCS3, were analyzed using STRING protein association networks. The results revealed that hepcidin was closely related to iron balance-related proteins and co-expressed with FPN1 and TFR1 (Figure A2A). A model map for the mechanism of hepcidin, FPN1, TFR1, and FTN regulating iron balance was constructed on the basis of our analysis results. The model map showed that hepcidin could initially bind to and degrade FPN1 and then control the iron concentration of the body (Figure A2B).

## 3. Discussion

Among mammals, humans express one hepcidin gene, namely, HAMP, whereas mice express two hepcidin genes, namely, HAMP1 and HAMP2 [29], but the murine HAMP1 and HAMP2 are in tandem and have likely arisen from a duplication in chromosome 7.HAMP1 is the main gene that participates in iron balance and immune response. The concentration of human HAMP in the serum is influenced by anemia associated with chronic kidney disease, inflammation, and impaired renal clearance [30]. In fish, HAMP1 is present primarily in the liver and is an ortholog of the mammalian sequences. However, the HAMP2 sequence is only found in acanthopterygians and exists in several tissues at a low concentration [31]. In *D. labrax*, HAMP1 is upregulated in response to iron overload and infection and downregulated during anemia and hypoxic conditions, whereas HAMP2 does not respond to either iron overload or anemia but is highly upregulated during infection and hypoxia [32,33]. In the present study, one type of hepcidin similar to HAMP1 in mandarin fish was cloned. The *Sc-hep* gene structure is phylogenetically conserved in vertebrates and has three exons and two introns, but the *Sc-hep* gene is smaller than the human hepcidin gene because of the small introns. Such small introns may reflect the size of the genome or different regulatory mechanisms of gene expression [34].

Similar to that in humans, the hepcidin gene from *D. rerio* [21], *C. idellus* [22], *S. aurata* [23], *P. crocea* [24], *D. labrax* [25], *T. fasciatus* [26], *B. lenok* [27], and *S. trutta* [28] have been cloned and proven to be mainly expressed in the liver. Hepcidin expression can be regulated by different biological mechanisms. DNA methylation of the CpG site in the promoter region can inhibit the expression of many genes and is an important research subject in epigenetics [35]. For example, DNA methylation can downregulate hepcidin gene expression in carcinoma cells [36]. However, in the present study, five CpG sites distributed in the promoter region showed a high methylation rate and no differential methylation level among different tissues, so DNA methylation might not be the main regulatory pathway to control *Sc-hep* expression in the liver. miRNAs can inhibit the expression of target genes by binding to the 3’-UTR of target mRNAs [37]. miR-130a is upregulated during iron deficiency and targets ALK2 (BMP type I receptor) to suppress hepcidin synthesis [38]. miR-122 targets hemochromatosis and hemojuvelin genes, whereas the overexpression of these genes activates the mRNA expression of hepcidin [39]. In the current study, we found for the first time that miR-19c-5p has a direct targeted inhibitory effect on *Sc-hep* mRNA.

The promoter region of *Sc-hep* contains binding sites for some transcription factor, such as USF1, USF2, STAT3, HNF, CEBPA, CEBPB, and NFKB, which are conserved in human [40,41], mouse [42,43], zebrafish [44], and white bass [11]. In the current study, binding sites for some other transcription factors, such as GATA1, GATA3, GATA4, STAT1, and TFAP2A, were also predicted in the *Sc-hep* promoter region, suggesting additional control of the expression of *Sc-hep* by different signaling pathways. Related in-depth studies may help reveal the specific expression characteristics of *Sc-hep* in the liver.

Research on hepcidin function in mammals has mainly focused on regulation iron balance [45] and participation in immune responses [7]. Hepcidin resists pathogens by destroying their cell membranes and inhibiting cell wall synthesis, cellular respiration, and entry of nucleic acids or proteins into cells [27]. Hepcidin inhibits the activity of pathogens by binding to and interfering with the DNA of pathogens [46]. Hepcidin can also promote the secretion of inflammatory cytokines [7]. In fish, hepcidin can resist the invasion of pathogenic bacteria and has a broad-spectrum antibacterial activity. Hepcidin treatment can significantly improve the survival rate of *C. idellus* infected with *Flavobacterium columnare* [22] and *T. fasciatus* infected with *Vibrio anguillarum* [26]. *P. crocea* hepcidin exhibits strong resistance to many pathogens, such as *Aeromonas hydrophila*, *Vibrio parahaemolyticus*, *Vibrio alginolyticus,* and *Vibrio harvryi* [24]. The mRNA expression levels of hepcidin in *B. lenok* [27] and *S. trutta* [28] significantly increased after they were infected with *Aeromonas salmonicida* and *A. hydrophila*. *Platichthys stellatus* hepcidin can inhibit the activity of *V. parahaemolyticus* and *Edwardsella tarda* [47].

The mechanism by which hepcidin regulates iron balance in humans mainly involves internalizing and degrading FPN1 to block the output of intracellular iron, thereby controlling extracellular iron concentration and systemic iron balance [16,17]. FPN1 is a membrane protein that is the only known iron export protein in vertebrate cells [13]. TF is a protein that exhibits high affinity to iron and transports iron into body parts where iron is needed and stored. TFR1 is a cell surface receptor of TF, which is the major protein that regulates iron uptake in most cells, allowing iron to enter the cells through endocytosis [14]. When iron demands in the body are high, intracellular iron is rapidly transferred across the BLM by FPN1. When the demand is low, intracellular iron can be stored in FTN, which consists of an apoprotein shell of 24 light- and heavy-chain subunits, surrounding a core of up to 4500 iron atoms [15]. Therefore, in our study, when the iron content of the body is excessive, the expression of Sc-hep in the liver significantly increases, indicating its importance for regulating iron balance, and the expression of FPN1 in the liver decreases, indicating that the iron output is blocked. TFR1 expression in the liver also decreases, indicating a decreased cellular iron uptake. FTN expression increase in the liver indicates that a sufficient amount of iron is stored in FTN in the cells. These results suggest that Sc-hep and human hepcidin may have similar functional mechanisms.

Iron metabolism and immunity are closely related. When the body is infected or inflamed, hepcidin levels in the serum are increased and iron concentration is decreased [45]. Hepcidin can limit the amount of iron required for microbial growth and survival by inhibiting iron release from macrophages [48].

Domestication affects the expression of hepcidin1 in liver, which is low in fast-growth strain and high in slow-growth strain. Domesticated rainbow trout may reduce innate immunity and change iron balance, involving more iron in hemoglobin synthesis [49]. Currently, there is no report on the relationship between dietary changes of fish and liver hepcidin. In this study, high expression of the *Sc-hep* gene through short-term domestication may be related to immune stress of *S. chuatsi*. IL-1β, IL8, TNF-α, and TLR22 expression increased after LPS injection. FPN1 expression also increased, and TFR1 expression decreased in the liver, indicating that pathogens that infected the host stimulated the differential expression of iron balance-related genes. These results showed that cytokine expression level and iron balance-related genes were affected when the host was attacked by pathogens. *Sc-hep* expression dramatically changed under different physiological conditions, indicating that this gene might serve as a biomarker of mandarin fish liver.

## 4. Materials and Methods

### 4.1. Fish and Sample Preparation

Mandarin fish were obtained from Guangdong tilapia fish farm (Panyu, Guangdong Province, China). *Cirrhina mrigala* fry was used as a live prey fish in this study. The fish larvae were cultured in different cement ponds (5 m × 3 m × 1.5 m) with a continuous water filtration system 2 weeks after hatching. The first group was continuously cultured with the live prey fish and served as a control group (PF group). The second group was fed artificially domesticated fish (AF group). In this group, 20 days after hatching, the fish in the AF group were trained to accept dead prey fish. Then, 40 days after hatching, they were trained to accept artificial diet gradually in 2 weeks.

Twenty healthy and uniformly sized fish from the PF group were selected 90 days after hatching and divided into four small groups with five fish in each group. Group 1 was injected with PBS (pH 7.4) and used as the control. Group 2 was injected with FS (Sangon Biotech, Shanghai, China, 30 mg/kg of body weight [BW], FS heptahydrate dissolved in PBS). Group 3 was injected with LPS (Sigma Chemical Co., St. Louis, MO, USA, 0.04 mg/kg of BW, LPS dissolved in PBS;). Group 4 was injected with Poly I:C (Sigma Chemical, 0.04 mg/kg of BW, Poly I:C dissolved in PBS). Five fish of each group were used for tissue isolation and RNA extraction 24 h after injection. The fish were euthanized. Tissue samples, including liver, intestine, kidney, head kidney, gill, stomach, and brain, were isolated, immersed in RNAlater solution (Ambion, Austin, TX, USA), and frozen at −80 °C for subsequent use. In one pond of the PF group, some fish appeared to have a disease in accordance with the method recommended by Office International des Épizooties [50]. The results revealed that they had ISKNV infection. The tissues of five fish were also isolated as previously described. All animal experimental procedures were performed in accordance with the Regulations for the Administration of Affairs Concerning Experimental Animals approved and authorized by the State Council of the People’s Republic of China and the Animal Ethics Committee of Shanghai Ocean University (2016NO. 4). Fish were sacrificed, and all efforts were exerted to minimize suffering. Clove oil (30–40 mg/L) was used for anesthesia.

### 4.2. Total RNA Extraction and cDNA Synthesis

Total RNA was extracted using TRIzol reagent (Invitrogen, Carlsbad, CA, USA) in accordance with the manufacturer’s instructions. Approximately 1 μg of RNA from liver, intestine, kidney, head kidney, gill, stomach, and brain was reverse-transcribed into cDNA by using a PrimeScript^TM^ RT kit (TaKaRa, Tokyo, Japan). miRNA first-strand cDNA in the liver was synthesized using a tailing reaction kit (Sangon Biotech). The obtained cDNA products were prepared for subsequent real-time quantitative PCR (qPCR) experiments.

### 4.3. DNA Extraction and Identification of Sc-Hep DNA Sequences

DNA was extracted using a TIANamp Marine Animals DNA kit (Tiagen Biotech Co., Ltd, Beijing, China) in accordance with the manufacturer’s instructions. The full-length mRNA and DNA sequences of *Sc-hep* were obtained from the full-length transcriptome (Accession number: SRR9649372) and genome (Accession number: PRJNA552957) of mandarin fish submitted to the NCBI database. One pair of primers (Sc-hep-F and Sc-hep-R) was designed to amplify the *Sc-hep* transcript and to verify the sequencing data. Two pairs of primers (Sc-hep-DNA-F1 and Sc-hep-DNA-R1; Sc-hep-DNA-F2 and Sc-hep-DNA-R2) were designed to amplify the *Sc-hep* gene. Eight genes, namely, *FPN1*, *TFR1*, *FTN*, *IL-1β*, *IL8*, *TLR22*, *TNF-α*, and *SOCS3* in mandarin fish were also cloned. All the primers are listed in Table A2.

### 4.4. Analysis of Sc-Hep Gene And Deduced Protein Sequence

A phylogenetic tree of the selected hepcidins was constructed with the Maximun Parsimony method by using MEGA7 based on sequence alignment with Clustal W [51]. A predicted secondary structure of Sc-hep was analyzed online with PredictProtein (https://www.predictprotein.org/). The presence and location of the signal peptide were predicted using SignalP 4.1 (http://www.cbs.dtu.dk/services/SignalP/). A predicted tertiary protein structure of Sc-hep was obtained using Phyre2 (http://www.sbg.bio.ic.ac.uk/phyre2) [52] and visualized using PyMOl. The promoter region of Sc-hep was analyzed in terms of transcription factor binding sites by using PROMO (http://alggen.lsi.upc.es/) [53] and JASPAR (http://jaspar.binf.ku.dk/) [54]. The online software STRING (https://string-db.org/) was used to analyze protein interaction.

### 4.5. qPCR Analysis of the Sc-Hep Gene

qPCR was conducted using SYBR Green Premix Ex Taq (Takara) in a CFX96 real-time PCR system (Bio-Rad, Hercules, CA, USA) as in our previous work [55]. β-actin was used as an internal reference. Gene expression levels were calculated using 2^−ΔΔCT^. In brief, reactions were carried out in a total volume of 10 μL containing 5 μL of 2× SYBR Premix Ex Taq, 1 μL diluted cDNA, and 4 μL of each primers (1μM). The amplification procedure consisted of an initial denaturation step at 95 °C for 2 min, and then 39 cycles at 95 °C for 5 s, 55 °C for 30 s, 72 °C for 30 s, followed by a final dissociation stage. Three technical replicates were set for each cDNA sample, and four biological replicates were set for each tissue sample to be tested. The expression levels were calculated by 2^−△△CT^ with △△CT = △CT_Sc-hep_ − △CT_β-actin_.

### 4.6. DNA Methylation Analysis of the Sc-Hep Promoter

DNA from the liver and the stomach was processed using an EpiTect^®^ Fast DNA Bisulfite kit (Qiagen, Duesseldorf, Germany) in accordance with the manufacturer’s protocol and then subjected to bisulfite sequencing PCR by using the methylated primers Sc-hep-met-F1, Sc-hep-met-R1, Sc-hep-met-F2, and Sc-hep-met-R2, which were designed with MethPrimer 2.0 (http://www.urogene.org/methprimer2/), to analyze the CpG sites located in the region between −899 bp and −1739 bp from the transcription start site (TSS). For bisulfite-treated DNA, the PCR reaction mixture was prepared using a TaKaRa EpiTaq™ HS kit (TaKaRa) in accordance with the manufacturer’s protocol. Four individuals were tested in each group, and 10 clones from each individual were selected for sequencing.

### 4.7. qPCR Analysis of miRNA and Dual-Luciferase Reporter Assay

The putative 3’-untranslated regions (UTRs) of *Sc-hep* mRNAs were used to predict miRNA target sites with RNA22 (https://cm.jefferson.edu/rna22/) [56] and miRanda (http://www.microrna.org/) [57]. The networks of these selected pairs were constructed using Cytoscape by defining the target pair interaction between *Sc-hep* and its target miRNA [58]. The relative expression levels of miRNA and *Sc-hep* were tested 24 h after FS or LPS injection. U6 was used as an internal reference. The miRNA and U6 downstream primers were universal primers (Universe R).

The 3′-UTR of wild-type (WT) *Sc-hep* was amplified from liver cDNA and cloned downstream of the firefly luciferase ORF in the pmirGLO vector (Promega, Madison, WI, USA) by using the XhoI and SalI restriction sites, obtaining the pmirGLO-3’-UTR-WT vector. For the mutated (MT) construct, the mutant 3′-UTR sequences of target genes, mutated in 5 bp in the conserved binding site, were synthesized and inserted into the pmirGLO vector, obtaining the pmirGLO-3’-UTR-MT vector. miRNA mimics were synthesized by GenePharma (Shanghai, China).

Three groups, namely, pmirGLO + mimics, pmirGLO-3’-UTR-WT + mimics, and pmirGLO-3’-UTR-MT + mimics, were transfected into HEK293T cells by using FuGENE^®^ HD (Promega) in accordance with the manufacturer’s instructions. Dual-luciferase assays were carried out 24 h after transfection by using the Dual-Luciferase Reporter Assay System (Promega) and Synergy2 (Bio-Tek, Winooski, Vermont, USA) Multi-Mode Microplate Reader in accordance with the manufacturer’s instructions. Firefly luciferase activity was normalized to Renilla luciferase activity by using Gen5 CHS 2.04 (Bio-Tek). Three replicate experiments were set for each group.

### 4.8. Statistical Analysis

Statistical analysis was performed in GraphPad Prism 7. Values were expressed as mean ± SEM. Student’s *t*-test was conducted to compare the difference in means between two groups; *p* < 0.05 was considered to be statistically significant.

## Abbreviations

Sc-hep	*Siniperca chuatsi* hepcidin
ISKNV	infectious spleen and kidney necrosis virus
LPS	lipopolysaccharide
Poly I:C	Polyinosinic–polycytidylic acid
FS	ferrous sulfate
PBS	phosphate buffered saline
PF	cultured with the live prey fish
AF	fed with artificially domesticated fish
BLM	basolateral membrane
HAMP	hepcidin
FPN1	ferroportin 1
TFR1	transferrin receptor 1
TF	transferrin
FTN	ferritin
IL-1β	interleukin-1 beta
IL8	interleukin-8
SOCS3	suppressor of cytokine signaling 3
TNF-α	tumor necrosis factor-alpha
TLR22	toll-like receptor 22
PPI	protein–protein interaction
C/EBP	CCAAT-enhancer-binding protein
HNF	Hepatocyte nuclear factor
NFKB1	Nuclear factor NF-kappa-B p105 subunit
USF	Upstream stimulatory factor
GATA	GATA-binding factor
STAT	Signal transducer and activator of transcription
TFAP2A	Transcription factor AP-2 alpha
NC	Negative control
WT	Wild-type
MT	Mutated-type

## Appendix A

**Table A1:** Transcription factor binding site prediction.

Transcription Factors	Putative Binding Site
TATA box	−32
CEBPA	−1738, −1619, −1500, −1102, −1024, −896, −790, −607, −136, −107
CEBPB	−1738, −1042, −896, −607, −136, −107
HNF1A	−1793, −1605, −201
HNF1B	−199, −330
HNF3A	−911
HNF3B	−1753, −1247, −597
HNF4A	−1158
HNF4G	−741, −1442
NFKB1	−164, −140
USF1	−1661, −1533, −1226, −783, −569
USF2	−1699, −904, −783, −655
GATA1	−1737, −1364, −986, −945, −321
GATA3	−943
GATA4	−324
STAT1	−288
STAT3 TFAP2A	−1548, −288 −1434

CEBP: CCAAT-enhancer-binding protein; HNF: Hepatocyte nuclear factor; NFKB1: Nuclear factor NF-kappa-B p105 subunit; USF: Upstream stimulatory factor; GATA: GATA-binding factor; STAT: Signal transducer and activator of transcription; TFAP2A: Transcription factor AP-2 alpha.

**Table A2:** Primers used in this study.

GenBank ID	Gene Name	Primer	Purpose
MK111643	*Sc-hep*-F	AAGGAGCTGACAAGAGTCAT	Clone the cDNA sequence of Sc-hep
	*Sc-hep*-R	GCTGCAACATTTTCTAATAAG	
	*Sc-hep*-qPCR-F	TGCTCACCTTTATTTGCCTTCA	Real-time quantitative PCR
	*Sc-hep*-qPCR-R	CGCTTCTGTCTGTTGTTATACG	
MK605396	*Sc-FPN1*-qPCR-F	CAGCCTCACCAGCGTTCTT	
	*Sc-FPN1*-qPCR-R	CTCCGACTCTATCACATTCTCCT	
MK605397	*Sc-FTN*-qPCR-F	TCCTACACCTTCCTTGCTCTG	
	*Sc-FTN*-qPCR-R	GACCACCTCTCCAATCCTCTC	
MK605398	*Sc-TFR1*-qPCR-F	GAATGCCTCTGCTGTGTTGAT	
	*Sc-TFR1*-qPCR-R	GCCTGTCGTGATGGTCTGA	
AY647430.1	*Sc-IL-1β*-qPCR-F	TTCAGTTCAGGAGGACGGATG	
	*Sc-IL-1β*-qPCR-R	TGTGGCAAGACAGGTAGAGATT	
JN180845.1	*Sc-IL8*-qPCR-F	GAAGAGCAGCAGAGTTATCATCA	
	*Sc-IL8*-qPCR-R	ATCTCAGTCTCCTCGCAGTG	
JN969981.1	*Sc-TLR22*-qPCR-F	CCTACGCCTACTACTTCTTCTTG	
	*Sc-TLR22*-qPCR-R	GGTCTTCCTGCTTCCATAGATG	
MK605399	*Sc-TNF-α*-qPCR-F	GCATACACAACCGCACTA	
	*Sc-TNF-α*-qPCR-R	CTTCCATTCCAGCACCAA	
KT895250.1	*Sc-SOCS3*-qPCR-F	TCCTCACCACACTCCACAAG	
	*Sc-SOCS3*-qPCR-F	CACATTGGATACGCAGGTTCTT	
FJ436084	*β-actin*-qPCR-F	GTGCGTGACATCAAGGAGAAG	
	*β-actin*-qPCR-R	GGAAGGAAGGCTGGAAGAGG	
	*Sc-hep-met*-F1	TGATGTGATTATATGTTAAGTATTTGTAAATATTA	Methylation
	*Sc-hep-met*-R1	ACTAATATATTAATACTATATATTTATACAAAC	
	*Sc-hep-met*-F2	ATTGTAAATGTTTTTTTAGATGTTGTTTTAATAG	
	*Sc-hep-met*-R2	ATCATCAAACTCAAAAATCTAAATACAAATAATAC	
	*Sc-hep-DNA*-F1	GGCAGGCTGTAGTTAATGTGT	Clone the DNA sequence of Sc-hep
	*Sc-hep-DNA*-R1	AGTTCTCCTGACTGATGGTTGA	
	*Sc-hep-DNA*-F2	CACACTCAACCATCAGTCAGG	
	*Sc-hep-DNA*-R2	CAGTTTCCCACTACAAATGTTATGC	
	*Sc-hep-3’-UTR-WT*-F	CTCGAGCAGTGTTGCAGTTGCAGTGGCCGT	qPCR analysis of miRNA and Dual-Luciferase reporter assay
	*Sc-hep-3’-UTR-WT*-R	GTCGACAATTTTGCAGGAAACATTAAATGAAC	
	*Sc-hep-3’-UTR-MT*-F	CTCGAGGGAGAGCTCATCATCATCGTCACTGAAGTGCAAGAGCTGGA	
	*Sc-hep-3’-UTR-MT*-R	GTCGACAACCGTACACGAAACATTAAATGAATCACTTCCTGTGTGC	
	*miR-196b*-F	TAGGTAGTTTCAAGTTGTTGGG	
	*miR-196d*-F	TAGGTAGTTTTATGTTGTTGGG	
	*miR-23b-5p*-F	GGGTTCCTGGCGTGCTGATTT	
	*miR-92b-5p*-F	AGGTGTGGGATGTTGTGCAGTGTT	
	*miR-19c-5p*-F	AGTTTTGCAGGATTGCATCCGG	
	*U6*-F	TTGGAACGATACAGAGAAGATTAGCA	
	*Universe*-R	GCTGTCAACGATACGCTCG	
